# Evaluation of the red cell hemolysis in packed red cells during processing and storage

**DOI:** 10.4103/0973-6247.75970

**Published:** 2011-01

**Authors:** R. N. Makroo, Vimarsh Raina, Aakanksha Bhatia, Richa Gupta, Abdul Majid, Uday Kumar Thakur, N. L. Rosamma

**Affiliations:** *Department of Transfusion Medicine, Indraprastha Apollo Hospitals, Sarita Vihar, New Delhi 110 076, India*

**Keywords:** Additive solutions, hematocrit, hemolysis, leucoreduction

## Abstract

**Introduction:**

Storage of red cells causes a progressive increase in hemolysis. In spite of the use of additive solutions for storage and filters for leucoreduction, some amount of hemolysis is still inevitable. The extent of hemolysis, however, should not exceed the permissible threshold for hemolysis even on the 42^nd^ day of storage.

**Study Design and Methods:**

Eighty units of packed red cells, 40 stored in SAGM post leucoreduction and 40 in ADSOL without leucoreduction filters, were evaluated for plasma hemoglobin by HemoCue Plasma Hemoglobin analyzer on the day of collection and on the 7^th^, 14^th^, 21^st^, 28^th^, 35^th^ and 42^nd^ days thereafter. The hemoglobin and hematocrit were also noted for all these units by the Beckman and Coulter analyzer. Percentage hemolysis was then calculated.

**Observations:**

Hemolysis progressively increased with the storage period in all the stored red cell units (SAGM as well ADSOL). However, on day 42^nd^ of storage, free hemoglobin in all the red cell units was within the permissible level (which is 0.8% according to the Council of Europe guidelines and 1% as per the US FDA guidelines). The mean percentage hemolysis was slightly higher in the SAGM-containing bags with an integral leucoreduction filter as compared to the bags containing ADSOL. However this difference was marginal and not statistically significant.

**Conclusion:**

Hemolysis of the red cells increases with storage. However, maximum hemolysis does not exceed the permissible limits at any time thereby indicating the effect of optimum processing and storage conditions on red cell hemolysis.

## Introduction

There is yet no substitute for human blood and its components. In recent years, the need for stricter control over the quality of blood and its products has been emphasized. One such quality indicator for stored red cell units is the extent of hemolysis.

Hemolysis represents the breakdown or disruption of the integrity of the red blood cell membranes causing the release of hemoglobin. Hemolysis of red cell units occurs during preparation of components as well as during storage, issue, and transport to the patient. Hemolysis in blood products is usually manifested by the presence of free hemoglobin in the red cell suspending media, such as plasma or additive solutions. Storage of red cells causes a progressive increase in hemolysis. The extent of hemolysis can be estimated by various techniques, visual assessment being the most common. Others include spectrophotometric assays, photometric method, and microplate technique.[1–4]

In spite of the increasing use of additive solutions for storage of red cells, some amount of hemolysis is inevitable. The extent of hemolysis, to some extent, varies with the composition of the additive solution used. ADSOL contains 50% more adenine and 150% more glucose in addition to 750 mg/dl of mannitol than does SAGM. The presence of leukocytes in the red cell units contributes significantly to an increase in red cell hemolysis during storage.[5–7] This is due to the release of various chemicals and enzymes, especially proteases from the leukocytes. For the same reason, various leukocyte reduction filters are commercially available. The presence of mannitol in the ADSOL medium reduces hemolysis even in the presence of leukocyte proteases. The percentage hemolysis progressively increases with the day of storage, irrespective of which additive solution has been used and whether or not the units are leucoreduced. However, even on day 42^nd^ of storage, free hemoglobin in any of the red cell units should not exceed the permissible level which is 0.8%, as per the Council of Europe guidelines[8] and 1% as per the US FDA guidelines.[9]

## Aims

The aim of the study was to evaluate the extent of RBC hemolysis with storage and the effect of leucoreduction and composition of the additive solution on the same.

## Materials and Methods

The present study on red cell hemolysis in packed red cells during processing and storage was conducted at the Department of Transfusion Medicine, Indraprastha Apollo Hospitals, New Delhi, between May and October 2009.

A total of 80 whole blood units were collected in CPD anticoagulant. After a holding period of at least 30 minutes, the components were separated from these blood units. Forty of the PRC units were stored in blood bags containing SAGM following 3 to 4 log leucoreduction via integral filters and 40 units were stored in ADSOL-containing bags, without integral filters giving only 1 log leucoreduction. All the packed red cell units were evaluated for plasma hemoglobin by HemoCue plasma hemoglobin analyzer. The first sample was collected immediately after processing, which was considered as the “Day 0” sample. Subsequently, all red cell units were stored under standard storage conditions at 2–6°C. Further evaluation was done on 7^th^, 14^th^, 21^st^, 28^th^, 35^th^, and 42^nd^ days of collection. The hemoglobin and hematocrit were also noted for all units each time by the Beckman and Coulter analyzer.

Each time 10 ml of the sample was collected aseptically from the units and centrifuged at 2000 rpm for 10 minutes. A large drop of the supernatant was pipetted out on the slide and the plasma/low Hb cuvette was filled from the drop. This was then inserted into the Hemocue photometer and the results were recorded. The percentage hemolysis was calculated by measuring the total Hb content, hematocrit, and supernatant Hb content of the PRC units using the following formula:

$$\mathrm{Hemolysis}\;=\;\frac{Plasma\;Hb\;\times \;\left(1-Hct\right)\;\times \;100}{B-Hb}$$

where Hb- hemoglobin; Hct- hematocrit; B-Hb- blood hemoglobin.

The data collected from the study were tabulated and then statistically evaluated to produce the following results.

## Results

**Figure 1:**
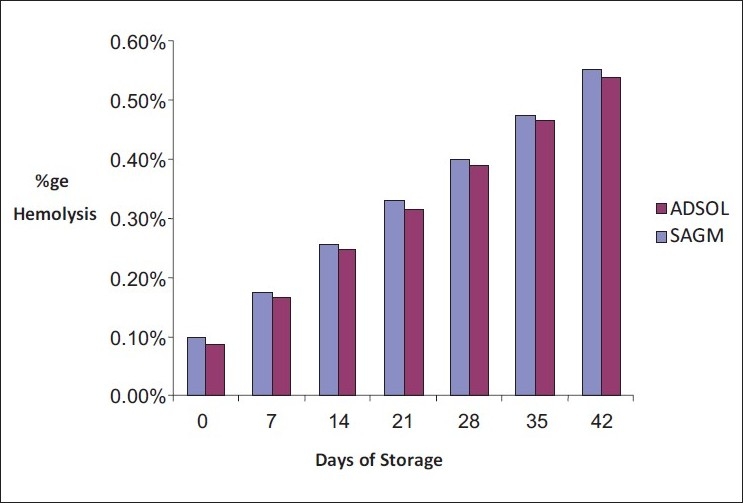
Comparison between SAGM and ADSOL

In the PRC units stored in SAGM, the least hemolysis was observed on the day of collection (mean = 0.1%), and the maximum hemolysis was observed on the 42nd day of storage (mean = 0.553%). Similarly, in the PRC units that were stored in ADSOL, an increase in the percentage hemolysis was observed from a mean of 0.087% on day 0 to 0.538% on the 42nd day [Tables 1 and 2]. The maximum percentage hemolysis shown by any sample was 0.710% in SAGM and 0.697% in ADSOL. It was also noted that the mean percentage hemolysis on each evaluation was slightly higher in 3–4 log leoucoreduced PRC units stored in SAGM as compared to those stored in ADSOL [Figure 1]. However, the difference was not statistically significant. None of the samples showed percentage hemolysis of more than 0.8%, which is the maximum acceptable limit of hemolysis as per the Council of Europe Guidelines.

**Table 1:** Hemolysis in SAGM-containing bags

Day after collection	Minimum hemolysis (%)	Maximum hemolysis (%)	Mean (%)
0	0.059	0.139	0.1
7	0.130	0.236	0.175
14	0.203	0.332	0.256
21	0.254	0.403	0.330
28	0.314	0.517	0.4
35	0.407	0.602	0.475
42	0.465	0.710	0.553

**Table 2:** Hemolysis in ADSOL-containing bags

Day after collection	Minimum hemolysis (%)	Maximum hemolysis (%)	Mean (%)
0	0.05	0.156	0.087
7	0.083	0.296	0.165
14	0.149	0.358	0.248
21	0.193	0.481	0.315
28	0.260	0.553	0.391
35	0.329	0.625	0.467
42	0.414	0.697	0.538

## Discussion

RBC units are prepared predominantly as concentrates (packed red cells) resuspended in additive solutions (SAGM/ADSOL) at our center. This prospective study was taken up to evaluate the extent of hemolysis during storage of packed red cells using two different additive solutions, and the effect of leucoreduction on the same. It was observed that the percentage hemolysis progressively increases from day 0 to day 42 of storage both in bags containing ADSOL (0.087–0.538) as well as SAGM (0.1–0.553) as additive solutions. It was also noted that the units that contained ADSOL as an additive solution showed slightly lower levels of hemolysis (maximum hemolysis being 0.697%) with respect to those containing SAGM (maximum hemolysis being 0.710%), even though the later were provided with integral leucoreduction filters. However, none of the units showed hemolysis more than the permissible limits, even on the 42nd day. At our center, we strictly follow the standard processing and storage guidelines. This could account for the satisfactory results obtained in our study.

Of the various methods that are available for the assessment of hemolysis, the visual method is the simplest; however the results are often inaccurate, misleading, and result in overestimation of hemolysis in red blood cells units.[2] In a study comparing spectrophotometric method and the photometric methods, Cardigan *et al*. demonstrated that both methods gave comparable results.[10] In our study we adopted the photometric method (HemoCue plasma hemoglobin analyzer) for the assessment of hemolysis.

Many authors in India as well as across the world have demonstrated that RBC hemolysis increases with the storage period of PRC.[11,12] In one such study, Sawant *et al*.[11] evaluated plasma hemoglobin levels using the tetramethylbenzidiene (TMB) method on day 1, 7, 14, 21, and 28 of collection. The hemolysis in all the PRC units increased with the day of storage. These findings were in confirmation with those in our study. Besides this, they also observed that hemolysis in units that were stored in SAGM was higher compared to the units stored in ADSOL. In another study, Zimmerman *et al*.[1] also observed slightly higher level of hemolysis in SAGM-containing bags. They attributed this increase to the preparatory technique which they used (the PRP method). Other authors believe that this difference in the level of hemolysis between SAGM- and ADSOL-containing bags is due to the difference in composition of the two additive solutions. ADSOL contains 50% more adenine and 150% more glucose in addition to 750 mg/dl of mannitol than does SAGM. Mannitol acts as a membrane stabilizer and dextrose is required for the metabolism of stored red cells.[11] This could also account for the lower levels of hemolysis in ADSOL-containing bags in our study. It has also been described by various authors that the presence of leukocytes in the red cell units contribute significantly to an increase in red cell hemolysis during storage,[7,10,11] and that prestorage filtration helps in reducing the extent of this hemolysis.[13,14] This is due to the release of various chemicals and enzymes, especially proteases from the leukocytes. For the same reason, various leukocytes reduction filters are commercially available. On the other hand, there have been a few reports in literature showing an increase in hemolysis with the use of leucoreduction filters.[15,16]

In our study, the difference in the level of hemolysis was higher in SAGM bags (with leucoreduction filters) than in the corresponding ADSOL-containing bags, but did not reach significance.

## Conclusion

Hemolysis is a very important parameter for assessing the quality of stored RBCs. On assessing the effect of duration of storage on red cell hemolysis, we concluded that there is a progressive increase in the percentage hemolysis with storage, irrespective of the additive solution used. However, the extent of hemolysis does not exceed the recommended limits at any time during storage, thereby indicating efficacy of the processing and storage protocol followed at our center. Further, no significant difference was noted in the extent of hemolysis between 3 to 4 log leucoreduced PRC units stored in SAGM and 1 log leucoreduced PRC units stored in ADSOL.
